# Morphological Neural Computation Restores Discrimination of Naturalistic Textures in Trans-radial Amputees

**DOI:** 10.1038/s41598-020-57454-4

**Published:** 2020-01-16

**Authors:** Alberto Mazzoni, Calogero M. Oddo, Giacomo Valle, Domenico Camboni, Ivo Strauss, Massimo Barbaro, Gianluca Barabino, Roberto Puddu, Caterina Carboni, Lorenzo Bisoni, Jacopo Carpaneto, Fabrizio Vecchio, Francesco M. Petrini, Simone Romeni, Tamas Czimmermann, Luca Massari, Riccardo di Iorio, Francesca Miraglia, Giuseppe Granata, Danilo Pani, Thomas Stieglitz, Luigi Raffo, Paolo M. Rossini, Silvestro Micera

**Affiliations:** 1 The Biorobotics Institute, Scuola Superiore Sant’Anna, Pisa, Italy; 2 Department of Excellence in Robotics & A.I., Scuola Superiore Sant’Anna, Pisa, Italy; 3 Department of Electrical and Electronic Engineering, Università di Cagliari, Cagliari, Italy; 4 Brain Connectivity Laboratory, IRCCS San Raffaele Pisana, Roma, Italy; 5 Bertarelli Foundation Chair in Translational Neuroengineering, Centre for Neuroprosthetics and Institute of Bioengineering, School of Engineering, École Polytechnique Fédérale de Lausanne (EPFL), Lausanne, Switzerland; 6 Department of Health Sciences and Technology, Institute for Robotics and Intelligent Systems, ETH Zürich, Zürich, Switzerland; 7 Institute of Neurology, Catholic University of The Sacred Heart, Policlinic A. Gemelli Foundation, Roma, Italy; 8 Laboratory for Biomedical Microtechnology, Department of Microsystems Engineering–IMTEK; Bernstein Center Freiburg and BrainLinks-BrainTools Center University of Freiburg, Freiburg, Germany

**Keywords:** Peripheral nervous system, Touch receptors

## Abstract

Humans rely on their sense of touch to interact with the environment. Thus, restoring lost tactile sensory capabilities in amputees would advance their quality of life. In particular, texture discrimination is an important component for the interaction with the environment, but its restoration in amputees has been so far limited to simplified gratings. Here we show that naturalistic textures can be discriminated by trans-radial amputees using intraneural peripheral stimulation and tactile sensors located close to the outer layer of the artificial skin. These sensors exploit the morphological neural computation (MNC) approach, i.e., the embodiment of neural computational functions into the physical structure of the device, encoding normal and shear stress to guarantee a faithful neural temporal representation of stimulus spatial structure. Two trans-radial amputees successfully discriminated naturalistic textures via the MNC-based tactile feedback. The results also allowed to shed light on the relevance of spike temporal encoding in the mechanisms used to discriminate naturalistic textures. Our findings pave the way to the development of more natural bionic limbs.

## Introduction

The sense of touch provides essential information about the external world. It can provide information about object characteristics during passive or active manipulation by means of the four classes of natural sensors embedded in the glabrous skin and in particular in the fingertips^1^. Thanks to their morphology and positioning, mechanoreceptors serve as the first processing stage of tactile interaction, implementing spatial and temporal filtering operations on the external stimuli^2^. Type-1 mechanoreceptors, namely Merkel and Meissner units, are located close to the epidermis and are known to encode fine localized details of tactile experience, whereas deeply-buried type-2 Pacini and Ruffini corpuscles have blurred borders and integrate the interaction over large receptive fields across the skin^3^. The classical Duplex theory, postulating the separate encoding via spatial cues of Merkel units for coarse textures and via vibrational signaling of Pacinian receptors for fine textures^4^, has been expanded by invoking unified peripheral neural mechanisms^5^ representing both coarse and fine stimuli via the integration of spatial and temporal information across the whole population of mechanoreceptors^6,7^.

Direct neural stimulation has been recently used in upper limb neuroprostheses for trans-radial amputees to successfully restore tactile information such as contact information^8^, and allowing force control, and shape and compliance recognition^9^. This opened up great opportunities to increase the overall usability^10^ of hand prostheses^11,12^. However, substantial efforts are still necessary to provide touch sensations closer to the natural ones^13^. In particular, both the physical characteristics of the physiological tactile sensors and their firing properties have to be replicated^14,15^ to create a more effective and natural connection with the peripheral nerves and therefore a more effective sensory restoration. This goal can be reached by exploiting the principle of “morphological computation”^16^ for neuroprosthetic applications, i.e., the embodiment of aspects of neuronal information processing into the physical structure of the system. The use of this morphological neural computation (MNC) approach can greatly improve the performance of the neuroprosthetic system without asking for a complex computational framework^17–19^.

We already explored the MNC approach in a previous work^17^ in which the tactile information collected by a biomimetic fingertip during passive stimulation was converted online into spike patterns mimicking mechanoreceptors activity, which were injected with TIME electrodes^20^ into the residual nerve fibers of the stump of one upper limb amputee. Relying exclusively on this stimulation, the amputee was able to rank pairwise the spatial period of fine gratings (0.5 to 3 mm) with very high accuracy.

Here we significantly extend these earlier results. First, we tested the sensitivity of tactile MNC to dynamically encode textures, with a particular attention to the dependency from sensors depth into the fingertip. Then, we showed that MNC enables trans-radial amputees to discriminate the complex textures of naturalistic materials, coherently with neuro-robotic studies^21^. Finally, we investigated which candidate neural codes might have provided to the nervous system the information leading to the successful discrimination, to shed light on how intraneural peripheral stimulations are processed by the amputees. This understanding will lay the ground for further advancements in the design of tactile feedback for upper limb neuroprostheses.

## Results

In this study, we show that the MNC-based peripheral neural stimulation delivered in trans-radial amputees using TIME electrodes^20^ allows to correctly identify naturalistic daily life textures with a performance very similar to the ones of healthy subjects. This sophisticated sensation was provided by injecting neuromorphic patterns of impulses into the residual nerves of two amputee subjects exploiting the use of biomimetic sensors able to gather also the shear component of stress arising during tactile exploration^22^ (see Methods and Supplementary Figs. 1 and 2).

**Figure 1:**
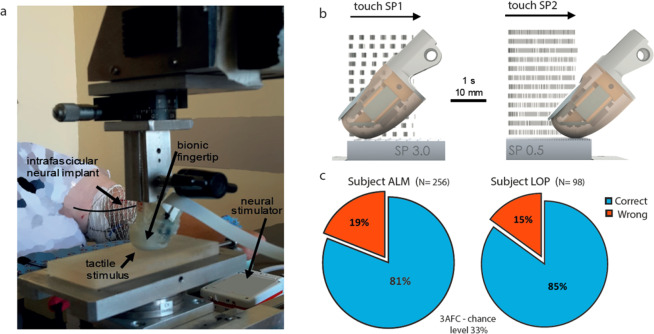
Neuromorphic artificial touch system encoding grating stimuli with type-1 mechanoreceptor model and performance of coarseness discrimination achieved by transradial amputees. (**a)** Experimental setup to evaluate the restoration of the perception of textural features in transradial amputees. (**b)** Transduction based on morphological neural computation (MNC) transforms the geometrical features of the surfaces into a sequence of spikes that are then delivered to the median or ulnar nerves by means of intraneural electrical stimulation. This specific illustration shows example patterns obtained with grating tactile stimuli with 3.0 mm SP1 and 0.5 mm SP2. Artificial fingertip touched the first half-grating for 2 s at 10 mm/s (20 mm sliding), followed by another tactile exploration of a second half-grating. Raster plots of the generated spikes are overlapped to the explored stimuli. (**c)** Overall performance achieved by the subjects in a three-alternative forced-choice experimental protocol (3AFC) of coarseness discrimination with the set of gratings described in Supplementary Table 1a.

We started from the results achieved in our previous study^17^, about the ability to restore tactile discrimination of gratings coarseness in upper limb amputees through intraneural tactile feedback with MNC. These results were confirmed with two additional trans-radial amputees (ALM and LOP, see Methods and Fig. 1) via stimulation of the median or ulnar nerve (Supplementary Fig. 3). We used the same set of stimuli as in the previous work: four gratings with two sides characterized by different values of Spatial Period (SP) of alternating ridges and grooves^17^ (Supplementary Table 1a). The interaction of a biomimetic fingertip with the surface of these gratings was transduced through MNC in bursts of spikes with regular inter-burst intervals that encoded the ratio between the shear exploratory velocity and the SPs of the stimuli. The amputees were able to correctly determine the coarsest side of the surface (81% for subject ALM, 85% for subject LOP, Fig. 1).

**Figure 2:**
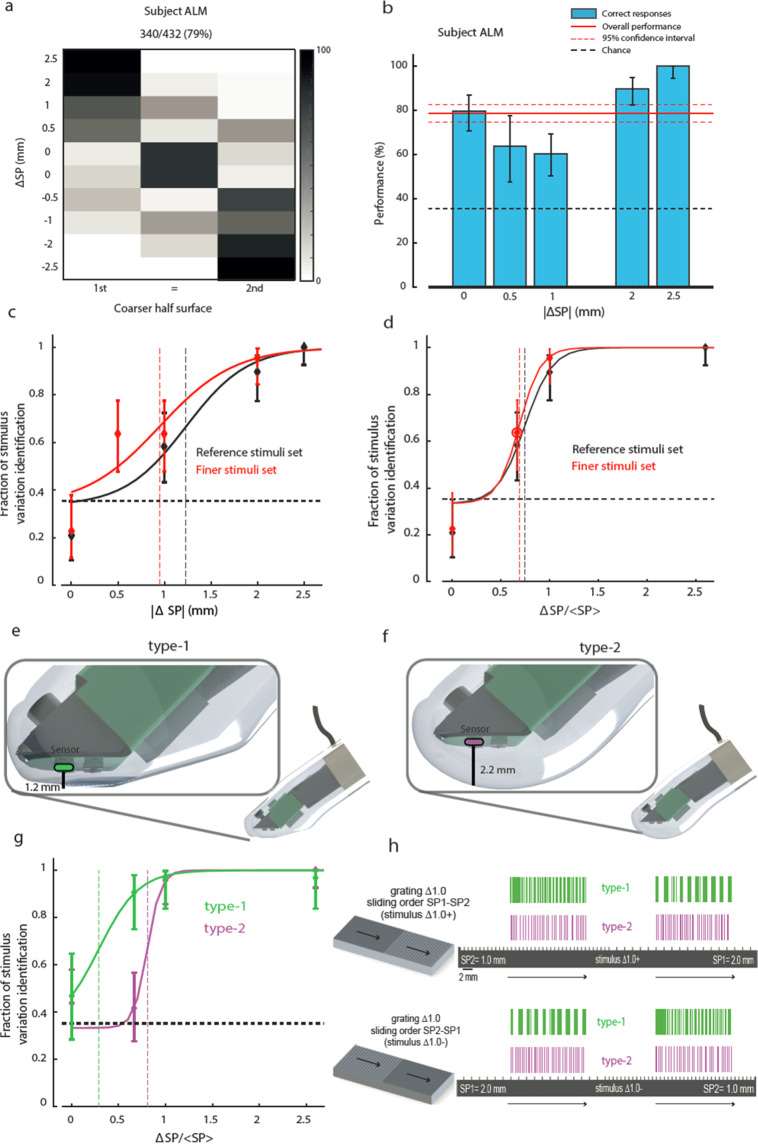
Psychophysical performance in 3AFC discrimination of grating coarseness, scaling with relative variation of spatial period, and comparison between type-1 and type-2 receptor models. (**a**) Confusion matrix of 3AFC psychophysical task. (**b**) Confidence interval of discrimination for the ΔSP of each grating. (**c)** 3AFC psychometric curve as a function of ΔSP for the reference set of stimuli (black) and for finer set (red). Vertical dashed lines indicate 3AFC perceptual thresholds as determined by the logistic fit. (**d)** Same as (**c**), but sorting the stimuli based on relative variation of spatial period ΔSP/SP. (**e,f)** Structure of the biomimetic fingertip with sensor location mimicking location of type-1 **(e)** and type-2 **(f)** mechanoreceptors. (**g)** Same as (**d)**, comparing the psychometric function resulting from the encoding of the spatial period via a type-1 receptor model (green) to that obtained via a type-2 receptor model (cyan). Note the shift in 3AFC perceptual threshold due to the change of the receptor model. (**h**) Spike trains generated by the same texture in the two directions during sliding of type-1 (green, top) and type-2 (cyan, bottom) mechanoreceptor.

In order to test the generality of our results to the variation of the stimuli set, we also presented subject ALM with a second set of gratings. Three out of four gratings were identical to the first set, but the grating with the largest difference in spatial period between the two sides (ΔSP) was replaced by a grating having a particularly fine ΔSP (SP = [0.5 1] mm, Supplementary Table 1b). Coarseness differences of gratings presented in both sets were decoded with the same performance, showing a lack of context effect. Instead, the discrimination performance of the grating with SP = [0.5 1] mm was not significantly different from the performance with the grating with SP = [1 2] mm (Fig. 2a,b). For both stimuli, the fractional variation of the spatial period was 100% while the ΔSPs were 0.5 mm and 1 mm, implying that the discrimination process could capture the relative rather than the absolute difference in SP between the two sides (Fig. 2c, compare with Fig. 2d). This suggests that subject’s discrimination might rely on the relative difference between the Inter Burst Intervals (IBIs) of the spike patterns transduced, which scales with the relative difference in SPs (Supplementary Fig. 4) rather than with the absolute difference between the IBIs^17^. This finding also accounts for previous results^17^ in which relative and absolute differences had the same ranking.

To challenge the MNC hypothesis, we also developed a second biomimetic fingertip with the sensors located deeper into the fingertip structure (Fig. 2e,f, see Methods and Supplementary Fig. 2). Given the presence of the artificial skin, this change in location could emulate to some extent the enlarged receptive field of deeply located receptors in comparison to that of corpuscles located close to the outer layer of the skin^23^. Coherently with expectations, with the reference stimuli set this design led to a diminished sensitivity toward finer differences in coarseness (for ΔSP/SP = 2/3 performance was 91% with surface-located receptor model and 42% with deeply-located sensor, p < 0.05, Clopper-Pearson exact interval; Fig. 2g). This confirms the possible role of surface-located tactile units to transduce the vibrational patterns of shear stress and thus encode the fine differences among the spatial periods of the physical stimuli into the temporal structure of the injected patterns (Fig. 2h). All the experimental sessions described in the following were performed with MNC based on surface-located tactile sensors.

While the discrimination of gratings spatial period is an encouraging result, naturalistic textures not only have much more complex patterns on the surface, far from being describable by a single parameter, but also differ for the properties of the material, e.g., surface roughness, compliance, and frictional properties. In order to explore whether our approach could enable the patients to recognize such rich stimuli, we finally tested whether the MNC-based encoding could allow the identification of naturalistic textures.

**Figure 3:**
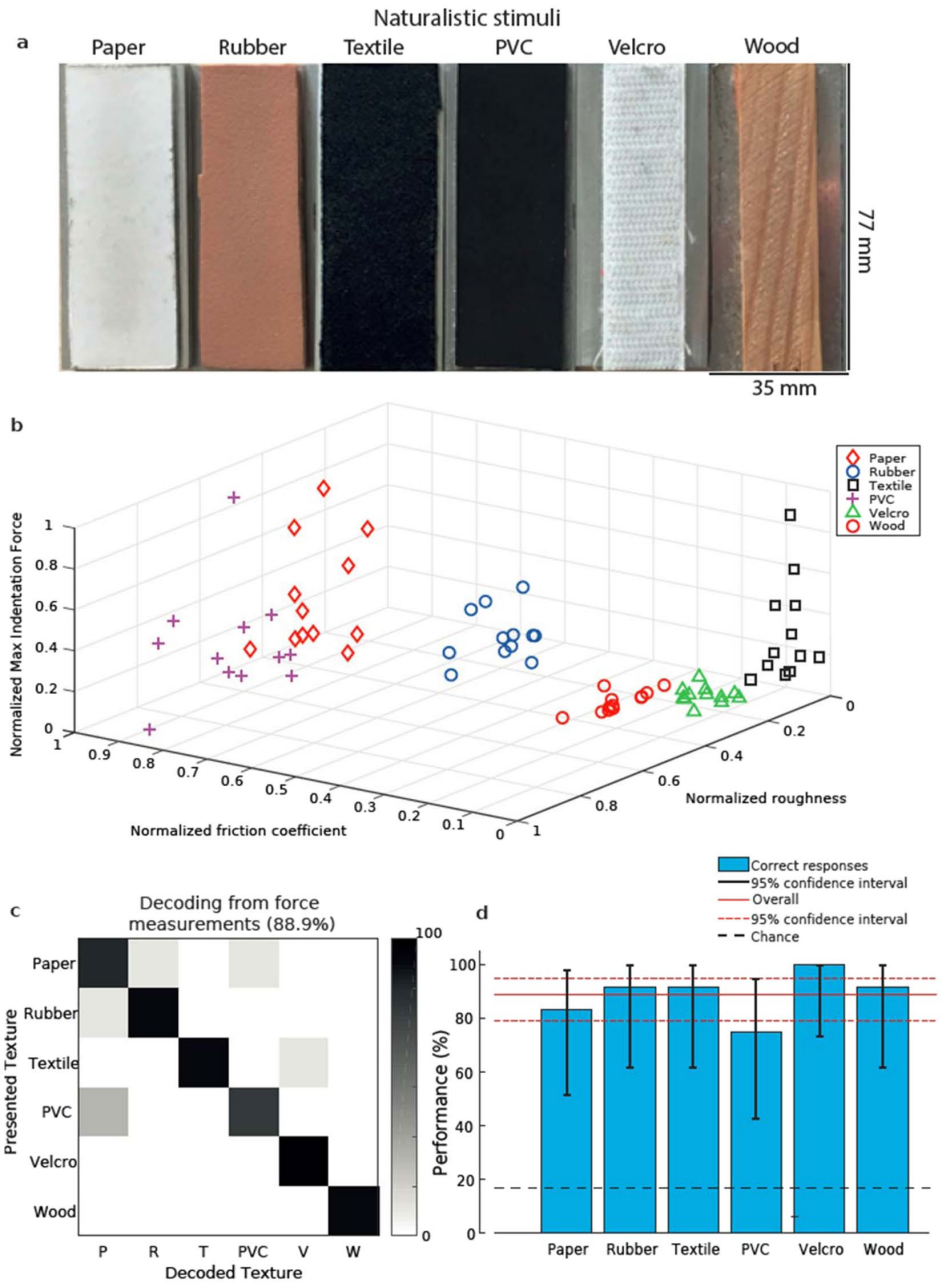
Frictional and roughness characteristics of the experimented naturalistic textures. (**a**) Picture of naturalistic textures (**b**). 3D scatter plot of normalized roughness, friction coefficient, and max indentation force for each presentation of each of the naturalistic textures. (**c**) Confusion matrix of kNN decoding of textures based on the aforementioned features. (**d**) Average performance and confidence for single-texture and overall decoding.

Six different materials were presented (see Fig. 3a and Methods). Note that although the trials involved passive indentation and sliding over the surface as in the sessions with gratings presentation, the experimental design for naturalistic textures identification was not a discrimination task but a classification task, in which each texture is presented individually and must be identified by the amputee (see Methods for details).

The selected textures were characterized by different properties, evaluated dynamically by the sensors through force components: surface roughness, maximal indentation force, and friction coefficient (Fig. 3b, see Methods for details). Notwithstanding the inherent experimental variability, it was possible to correctly identify the presented texture knowing only the aforementioned features of the normal and shear force components, as shown by the decoding performance (88.9%, with each single texture correctly identified far above chance, see Fig. 3c,d). The question is then (i) whether this information (and possibly extra information) is conveyed by spike patterns generated through type-1 MNC, (ii) whether the nervous system of the patient is able to extract salient information from spike patterns.

During the sliding phase, all the textures induced irregular and complex patterns of spiking activity (Supplementary Fig. 5). Features of spike patterns were indeed associated to the aforementioned specific properties of the textures: the average spike count during sliding significantly correlated with friction (p < 0.00001), the coefficient of variation of the interspike interval during sliding significantly correlated with texture roughness (p = 0.005), and the average spike count significantly correlated with maximal normal force during indentation (p = 0.003), as shown in Supplementary Fig. 6. This indicates that these neural features of the MNC spike patterns could be exploited to properly classify the different naturalistic textures.

**Figure 4:**
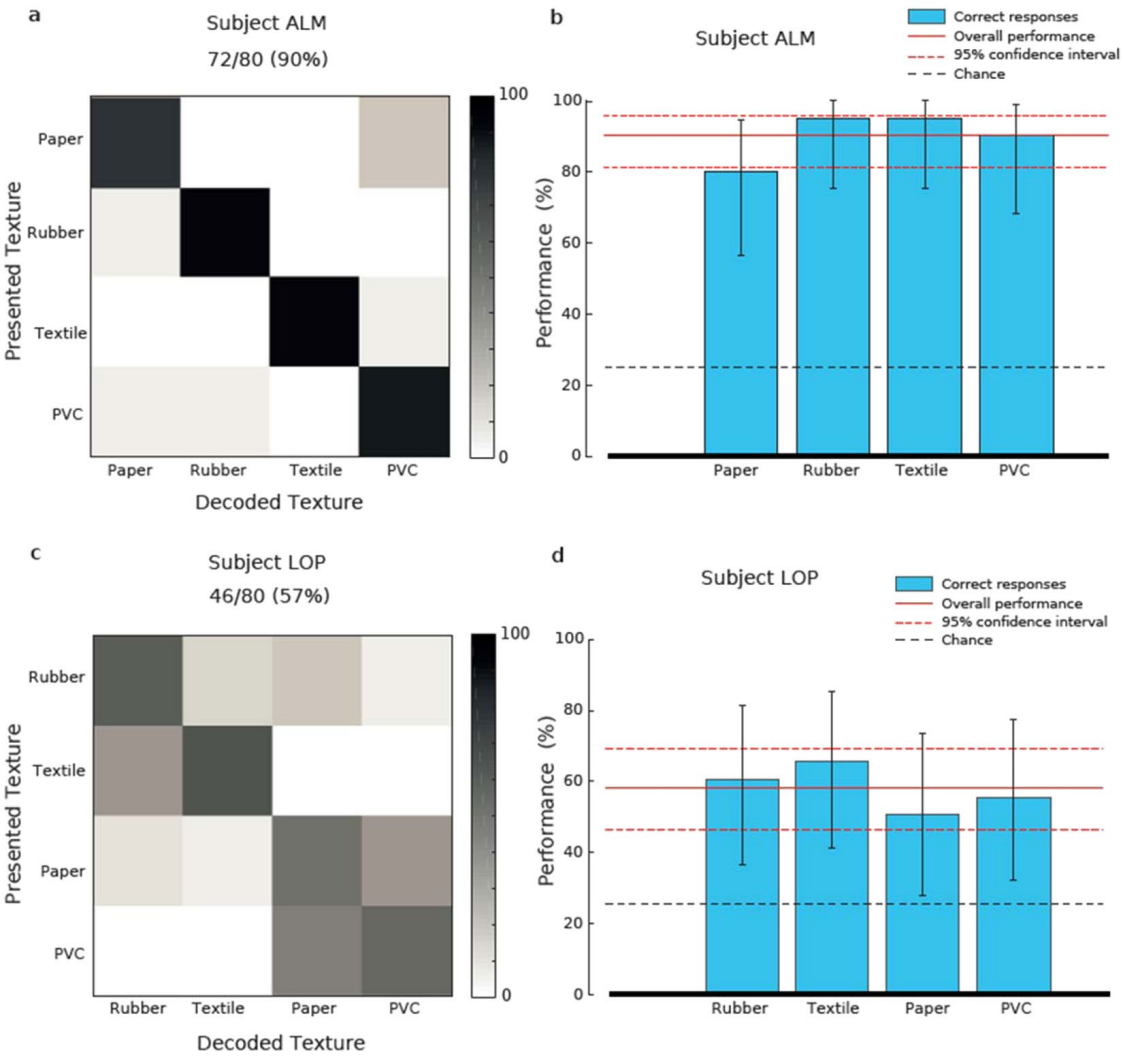
Identification performance with naturalistic textures. (**a)** Confusion matrix of subject ALM performance in the identification of 4 naturalistic textures. (**b)** Confidence interval of identification of the same textures as a. (**c,d)** Same as a-b, for subject LOP.

**Figure 5:**
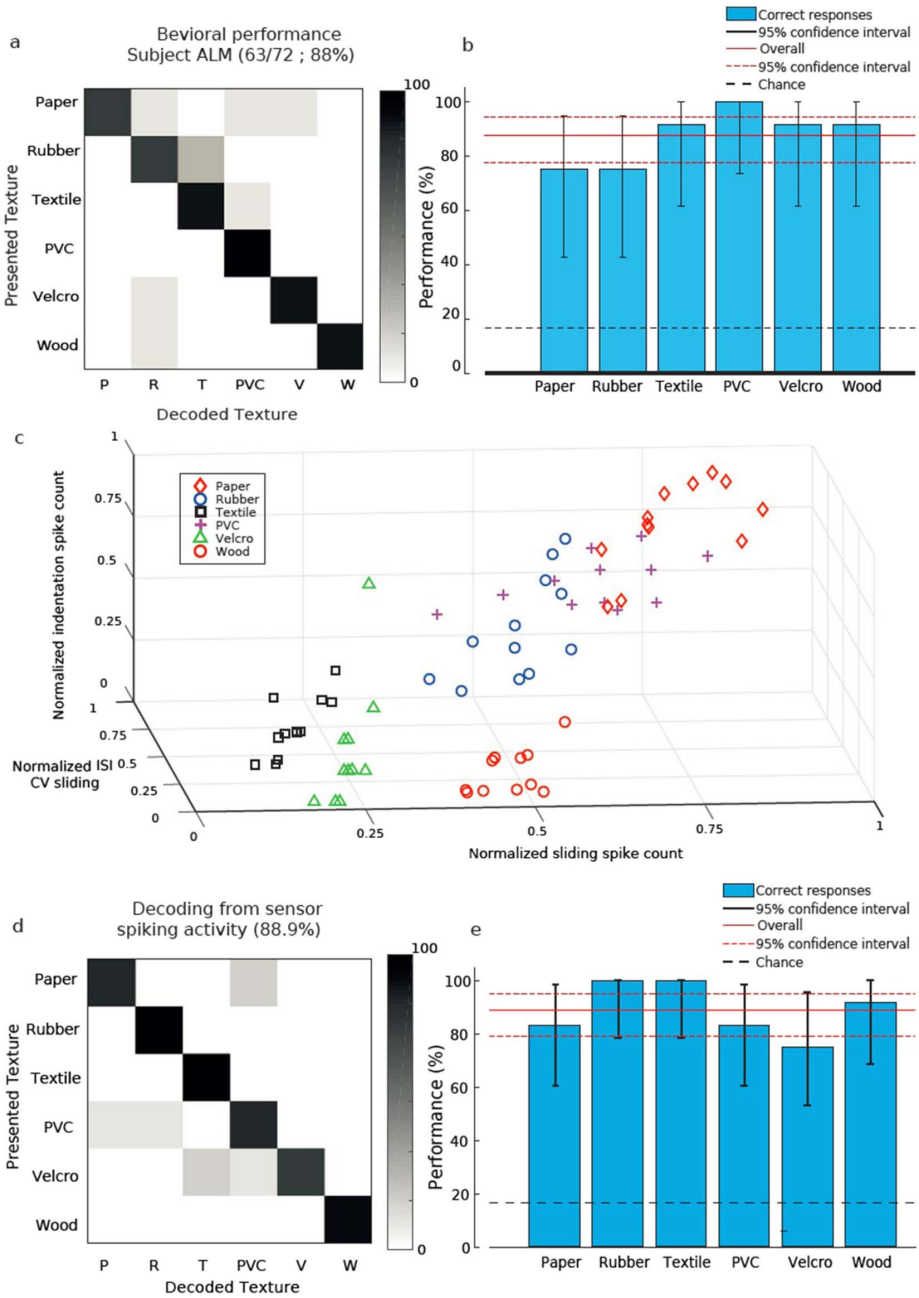
Analysis of candidate decoding mechanisms for discrimination of naturalistic tactile stimuli. (**a,b)** Same as 4a-b, extended to 6 naturalistic textures. (**c)** Scatter plot of neural features related to texture properties for all presentations of all textures. (**d,e)** Performance of a decoder using kNN clustering of the spike trains based on neural features of **(c)**, represented as confusion matrix **(d)** or displaying the confidence interval for all textures **(e)**.

Both subjects were indeed able to identify a set of four textures (90% subject ALM, 57.5% subject LOP see Fig. 4a,c), with each single stimulus being discriminated significantly above chance level (Fig. 3b,d; p < 0.05 Clopper-Pearson exact interval). The performances of the two trans-radial amputees were compatible with those of intact subjects (Supplementary Fig. 7), both in the overall performance and in the asymmetric confusion matrix. In particular, ALM performance was close to the best performances of healthy subjects while LOP performed similarly to the worst performances of healthy subjects. Consequently, subject ALM was also presented with a set of six textures and was able to identify them (Fig. 4a), with very good performance for each texture (Fig. 5a,b; p < 0.05 Clopper-Pearson exact interval) and discrimination ability similar to that of healthy subjects (Supplementary Fig. 7).

We investigated then more closely which aspects of the injected spike trains could form the basis for such decoding performance. Previous studies on tactile perception^7,13^ suggested that the temporal structure of the neural activity in the somatosensory pathway plays a pivotal role in this kind of tasks. Briefly, in a spike count code the neural feature conveying information is only the total number of spikes fired during the stimulus delivery, while in a temporal code there is also a significant amount of additional information in the precise sequence of the spike times^24^. A temporal code carries at least the same amount of information than a spike count code. Several animal studies suggest that spike count codes are unable to carry the wealth of information coming from tactile inputs and that the somatosensory system consequently relies on temporal codes^25,26^. However, ultimate evidences supporting this model have not been provided on this respect since most studies in the field investigated reaction to vibratory stimuli or perception of regular spatial patterns which might lead to phase locking of responses^23^. Instead, in our study with naturalistic textures we presented highly complex structures that lead to an irregular temporal structure of the response which allowed to explore the relevance of spike temporal encoding. To this aim, we used a kNN decoder (see Methods for details) to classify the naturalistic textures using features extracted from the spike patterns transduced by the biomimetic finger mimicking type-1 receptors. Results were compared with the performance of ALM with six naturalistic textures, since it was the performance providing the most stringent comparison for candidate codes.

A decoding based on the spike count during presentation of different textures (see Methods) failed to account for the subject’s performance, as it achieved only a 73.6% performance, far below the one achieved by ALM (Supplementary Fig. 8). This result suggests that there might not be enough information in the spike count to describe the behavioral performance of ALM. This hypothesis was further investigated in two steps.

First by computing the confusion matrix information (CMI, see Methods and^27^) associated to subject ALM responses. We found that the CMI was 1.94 bits. The features used by ALM for the decoding are expected to carry at least the same amount of information. The amount of information carried by the spike count was instead only 1.46 bits. This result is independent from any assumption on the decoding procedure enabled by the nervous system of ALM and hence rules out the possibility that the observed ALM performance was achieved using a spike count code. This supports the hypothesis that the subject was able to draw information from the temporal structure of the injected stimuli to achieve the behavioral result observed.

The we hypothesized that such temporal code could be based on the encoding of the physical features of the textures (Fig. 3) by the spiking activity (Supplementary Fig. 6), i.e., not only the spike count during slide considered above, but also by the coefficient of variation of the interspike interval during sliding (ISI CV) and the mean spike count during indentation (Fig. 5c). A kNN decoder based on these features achieved indeed a 88.9% performance (Fig. 5d,e). This performance is compatible with psychophysical results (compare Fig. 5a,d and see the overlap between the Clopper-Pearson confidence ranges in Fig. 5b,e). Combining only spike count and ISI CV achieved a similar decoding performance of 86.1% (Supplemental Fig. 8a–c). Note that no single feature could account individually for the decoding performance of ALM, as spike count during indentation carried 0.96 bits of information and the ISI CV 1.88 bits, both below the information value of 1.94 of the patient’s decoding. The combinations of the features carry instead all more than 2 bits of information and are then compatible with behavioral results (spike count indentation and spike count sliding = 2.1 bits, ISI CV and spike count sliding = 2.2 bits, ISI CV and spike count sliding = 2.3 bits, all together 2.3 bits). Therefore, the decoding of this set of naturalistic textures could have been performed only by a combination of features.

The decoding procedure described above relies on reasonable assumptions about the features of the neural signal that could be extracted by the subject to perform the classification task. To evaluate the decoding performance of an assumption-free decoding of the injected temporal patterns of impulses, we classified them also based on Victor Purpura spike train-distance (VPd)^28^ over a broad range of timescales^21^. Very long (>850 ms) or very short (<12 ms) integration times were found not to be compatible with subject performance (Supplementary Fig. 8). Focusing instead on a 100 ms timescale the overall VPd-based decoding performance was 93.1%, with a performance compatible with psychophysical results (Supplementary Fig. 8). These results are not conclusive about the specific features of the injected spike train used by the subject to decode textures, but they show that a spiking temporal code could underlie the strategies implemented every day to gather textural information from daily life objects.

One possible limitation of our MNC was that the injected patterns emulated the activity of a single mechanoreceptor, and that the biomimicry was not truly natural: in our experiments both the MEMS sensors located close to the skin surface (1.2 mm, Supplementary Fig. 2a) and the deeper ones (2.2 mm, Supplementary Fig. 2b) were integrated on a bone-like rigid support covered by soft polymeric material, while in the real finger all mechanoreceptors float in the surrounding soft tissue. To explore the limits of our approach, we simulated then with a sophisticated neurophysiological model^14^ the response of a large and diverse population of mechanoreceptors to the same tactile stimulation applied to the fingertip of the hand (Supplementary Fig. 9a). Decoding performance with the VP approach was very high (Supplementary Fig. 9b) and compatible with the one achieved using the same approach on the spike trains injected during experimental session. This indicates that, at least for this particular task, our MNC approach captured most of the information available for naturalistic textures discrimination.

## Discussion

Neuroprosthetic technologies can provide important clinical benefits to disabled persons^29^, and at the same time can provide a unique opportunity to validate neuroscientific hypotheses thanks to the possibility to chronically get direct access to the neural system of human subjects. Our study represents a combination of these two advantages. In fact, mimicking the embodied intelligence of biological sensors through morphological neural computation allowed the two subjects to successfully discriminate naturalistic textures and at the same time shed new light on the temporal mechanisms underlying textural discrimination. While presenting an efficient technical approach to provide tactile sensory feedback to upper limb amputees, we also hypothesized different candidate neural mechanisms that might underlie the achieved textural classification. Spike count could not account alone for the observed performance, suggesting that a temporal code is required, at least in the form of an estimate of the regularity of the firing patterns (as for ISI CV). In particular, the code displayed in Fig. 4 is not only grounded in actual material properties, but was indirectly suggested by patient ALM that, after the end of the experimental sessions, told us that she was recognizing the textures by collecting “information about softness during indentation and information about regularity during sliding”. Interestingly, our results achieved during (controlled) passive touch experiments could be generalizable to active touch activities since several evidences suggest a strong similarity between the two cases from a neurophysiological viewpoint^30^. An open issue in this respect will be about how to allow the subject to decouple velocity-related compression or dilation of temporal pattern of the neural stimulation^7,21,31^.

Our findings provide also useful indication from a technological viewpoint. In fact, so far the most advanced neuromorphic touch technologies focused on encoding into spikes the normal component of stress/strain arising due to physical interaction^18,19,32,33^. Instead, in our present study we advance the technological state of the art by exploiting the shear transduction capabilities of our biomimetic sensor (Supplementary Fig. 1). The comparison with the decoding performance achieved by a very sophisticated neurophysiological model^14^ based on the simulated response of a large population of natural touch sensors sensitive only to normal components show how gathering also shear stress information could allow to get good discrimination abilities with a very limited number of sensing elements as it is in our case.

### Limitations and perspective

The main limitation of our study is the reduced number of subjects and the variability between them. Even though further tests will be needed to assess whether the average performance is closer to the high end of patient ALM or to the low end of patient LOP, we must stress that both performances are not only significant but also within the range of those achieved by the control test of intact subjects.

One technical limitation of our conclusions regarding MNC is instead that we implemented type-2 mechanoreceptors without changing the sensor type but only locating it deeper into the fingertip skin. A future step would be to tune the properties of the mechanoreceptor model to match more closely neurophysiological data. Our sensor encodes tactile interaction integrating slowly-adapting and fast-adapting information in a single device, particularly for the surface-located positioning. The concurrent encoding of textural information by multiple receptor types is consistent with integrated models proposed in the literature^7^. It is remarkable to show that in such integrated framework proposed by Weber and Bensmaia, Meissner corpuscles (that are type-1 receptors) have a very important role for fine textures coherently with the findings that we present with our artificial touch technology. Nevertheless, there is an apparent mismatch with respect to the previous findings with Pacinian corpuscles (that are type-1 receptors) and our deeply-located sensor. This is presumably because for our deep sensor positioning the filtering effect introduced by the higher thickness of the material causes a dominance of a slowly-adapting encoding (Ruffini-like, which are not investigated in the non-human primate study by Weber and Bensmaia) instead of fast-adapting one (Pacini-like, which encodes acceleration information correlated to textural features). Another limitation of our study, due to temporal restraints in the experiments involving human subjects, is the fact that we tested only a single sensing condition (400 mN indentation force and sliding speed of 10 mm/s). However, our previous study on the property of the biomimetic fingertip^21^ showed that for a broad range of forces and speeds the spike patterns carry enough information to allow for decoding of complex textures. It also showed that a decoding across speeds is possible if the instantaneous speed is known, e.g., if an efferent copy or proprioceptive information related to the movement performed is available. Another concurrent compensatory mechanism to unwrap the velocity dependence of the temporal patterns of neural stimulation could be based on parallel multichannel stimulation strategy, recruiting afferent fibers in a selective and coordinated manner^34,35^. Such biomimetic stimulation strategy would contain information able to reject velocity-variations of the stimulus via phase differences of neighboring mechanoreceptors. Furthermore, variations in indentation force do result in different spike patterns, but these are likely to be clustered in the following steps of tactile information processing.

In the next future, it will be possible to further extend the use of the MNC approach by integrating information from both type-1 and 2 mechanoreceptors to reproduce and possibly integrate a broader range of sensations. Moreover, it will be possible to exploit recent advancements in the developments of bioinspired sensitive skin^18^ and biomimetic models^14,36^ to provide sensation even closer to the natural touch with an increase benefit for disabled people.

## Materials and Methods

### Subjects recruitment and surgical procedure

Two amputees participated in the study. Subject 1 (ALM) was a 54-year-old female with a left wrist disarticulation incurred 23 years prior to the study. Subject 2 (LOP) was a 48-year-old female with a proximal left trans-radial amputation incurred 2 years prior to the study. Both subjects were enrolled for a period of about 6 months, during which experimental sessions were randomized. The data reported in this manuscript was obtained over a period of several days with both amputees.

Ethical approval was obtained by the Institutional Ethics Committees of Policlinic Agostino Gemelli at the Catholic University, Rome, Italy, where the surgery was performed. The protocol was also approved by the Italian Ministry of Health. Informed consent was signed. During the entire length of our study, all experiments were conducted in accordance with relevant guidelines and regulations. Informed consent for publication of identifying information/images was signed.

The surgical approach to implant TIMEs has been extensively reported elsewhere^9^. During general anesthesia, through a 15 cm-long skin incision on the left arm above the elbow, the median and ulnar nerves were exposed to implant a proximal and a distal TIME. Each TIME had 14 electrical active sites usable to deliver the electrical stimulation. Intraneural stimulation was injected via percutaneous wires. After 180 days, the four neural electrodes were removed, in accordance with the protocol and the obtained permissions.

### Sensorized finger

The core element of the artificial fingertip was a Micro Electro Mechanical System (MEMS) sensor with 4 transducing piezoresistors implanted at the base of a cross-shaped structure^37^.

Sensor data was sampled at 380 Hz per channel by a 24-bit Analog to Digital Converter (ADS1258, Texas Instruments, USA). The readout electronics were integrated onto a 3D printed bone of an artificial fingertip and then packaged with polymeric skin-like compliant material (Dragon Skin, Smooth-On, USA).

Using two different thicknesses of the soft skin-like cover, two versions of the artificial finger were designed, to emulate to some extent type-1 surface-located (1.2 mm depth, Fig. 2e and Supplementary Fig. 2a) and type-2 deeply-located (2.2 mm depth, Fig. 2f and Supplementary Fig. 2b) receptor positioning. The positioning of the sensors was characterized by means of industrial computer tomography measurements (by Pontlab srl with Baker Hughes GE v|tome|x m 300 kV machine).

### Mechano-neuro transduction

MEMS sensor data was acquired via SPI by a Field Programmable Gate Array (Zynq-7000 on sbRIO-9651, National Instruments) programmed by means of LabView FPGA. The data was then passed to the real-time layer of the same system on a module unit (ARM Cortex A9 on sbRIO-9651, National Instruments), which implemented an artificial mechanoreceptor model emulating the neural code being recorded during human microneurography sessions^38,39^. To this aim, the Izhikevich spiking neuron (Eqs. 1–3) was customized so to receive a signal transduced by the MEMS sensors embedded on the artificial fingertip (Eqs. 4, 5)^17,40,41^.

Input current of the spiking neuron model was obtained by subtracting (Eq. 4) sensor piezoresistive outputs (S_x+_ and S_x-_) belonging to opposite tethers of the cross-shaped structure, to obtain a half-rectified component (Eq. 5) highly correlated to the tangential component of force acting locally on the finger surface^42^.

The computed binary response constituted the output of the neuromorphic artificial touch system, which was triggered via a custom neural stimulator each time that the pulse corresponding to a spike had to be delivered.

1 $$\frac{{\rm{dv}}}{{\rm{dt}}}={{\rm{Av}}}^{2}+{\rm{Bv}}+{\rm{C}}-{\rm{u}}+\frac{{{\rm{I}}}_{{\rm{x}}}}{{{\rm{RC}}}_{{\rm{m}}}}$$

2 $$\frac{{\rm{du}}}{{\rm{dt}}}={\rm{a}}({\rm{bv}}-{\rm{u}})$$

3 $${\rm{if}}({\rm{v}}\ge {{\rm{v}}}_{{\rm{th}}}),\,{\rm{then}}\,\{\begin{array}{l}v\leftarrow c\\ u\leftarrow u+d\end{array}$$

4 $${{\rm{S}}}_{{\rm{x}}}={{\rm{S}}}_{{\rm{x}}+}-{{\rm{S}}}_{{\rm{x}}-}$$

5 $${{\rm{I}}}_{{\rm{x}}}=\{\begin{array}{c}K{{\rm{S}}}_{{\rm{x}}},\,x\ge 0\\ 0,\,x < 0\end{array}$$

The following parameters were used: A = 0.04/sV; B = 5/s; C = 140 V/s; C_m_ = 1 F; R = 1Ω; a = 0.02; b = 0.2/s; c = −65mV; d = 8 mV; v_th_ = 30 mV.

The approach adopted was hence different in comparison to those used in previous studies^7,43^, in which different features of the stimulus (position, velocity, acceleration, jerk) are extracted, processed, summed and then injected into the simulated mechanoreceptors. In this study, instead, a piecewise linear function of the raw output of the sensors was injected as input to the Izhikevich neuron model, and the processing of the temporal structure of the input was performed by the adaptive properties of the neuronal model. So, we did not explicitly take into account any temporal derivative of the stimulus, but the response of our simulated mechanoreceptors was sensitive to them.

### Neural stimulator

During the experiments, we used a custom-made stimulator called EARNEST, which is a prototypical, wearable, complete embedded platform for neural prosthetic applications realized at University of Cagliari^44,45^. The device includes multi-channel ENG recording and programmable electrical stimulation functionalities from up to 4 intra-fascicular electrodes with 16 channels each and EMG recording from 4 differential surface electrodes. The stimulating capabilities include the possibility to simultaneously use up to 64 stimulating channels. The device allows controlling amplitude, duration and shape of each single current stimulus to produce different stimulation profiles. EARNEST can generate either pre-configured electrical stimuli (such as pre-defined bi-phasic charge-balanced pulses) or user-defined current waveforms. Each stimulation channel can provide a programmable current of up to 512 µA, with steps of 10 µA resolution, a minimum pulse duration of 10 µs, an output voltage compliance of 17 V and a maximum pulse frequency of 1 kHz. Pulse generation is triggered by simple commands sent through a 4-wire Serial Peripheral Interface (SPI).

### Sensation mapping procedure

In order to test the sensory feedback efficacy in discriminating textures, a single active site was chosen for each subject eliciting a reliable sensation on the phantom fingertip. In particular, before each experimental session a specific active site was selected based on the sensation mapping procedure of the week (Supplementary Fig. 3c). Since each TIME electrode had 14 active sites (56 for four TIMEs), all sensations evoked by stimulating from each contact were characterized weekly.

In the mapping procedure, short trains of current pulses with variable intensity (current amplitude), duration (pulse width) or repetition frequency were delivered at least 5 times through every active site. Charge-balanced, biphasic, cathodic-first, rectangular stimulation pulses were applied versus a ground electrode integrated on the TIMEs.

The subjects were asked to report: the location, extent, type and strength (in a scale between 0 and 10) of the generated percepts whenever they perceived them. Using this data, it was possible to estimate the lower (thresholds) and upper (saturation) limits of the current intensity which are able to induce reliable sensations. Thus, a map of the sensations referred to the correspondent active sites was obtained.

When the evoked sensation map was acquired, a single active site was selected as the optimal candidate for the experiments in terms of sensation quality, intensity and location (Supplementary Fig. 3b).

### Psychophysical protocol of coarseness discrimination in gratings

The experiments in which the patients performed coarseness discrimination in gratings with different spatial periods (SPs) were based on a three-alternatives forced-choice psychophysical protocol^17^, with the subject declaring whether the first half-surface was perceived as having coarser, finer or same spatial coarseness in comparison to the second half.

The gratings were fabricated with 3D printed plastic material (Project HD 3000, 3D Systems) and had SP from a minimum of 0.5 mm to a maximum of 3.0 mm (Figs. 1b, 2).

The gratings were presented using a mechatronic platform^38^ in randomized sequences of 16 trials, each composed of 4 presentations of 4 surfaces.

In the reference stimuli set the surfaces had the following differences in SP between the two halves of the stimulus: ∆0.0 (SP1 = 1.5 mm; SP2 = 1.5 mm), ∆1.0 (SP1 = 1.0 mm; SP2 = 2.0 mm), ∆2.0 (SP1 = 0.5 mm; SP2 = 2.5 mm) and ∆2.5 (SP1 = 0.5 mm; SP2 = 3.0 mm), as in our previous study^17^.

In the finer stimuli set the stimulus with largest difference in coarseness ∆2.5 was substituted by a stimulus with ∆0.5 (SP1 = 0.5 mm; SP2 = 1.0 mm).

In each session, the 4 trials per each stimulus were composed of 2 trials with presentation of the two half surfaces in the SP1-SP2 order and 2 trials with presentation in the reverse order. This switch does not affect the texture with the same coarseness in the two half surfaces. Overall then we have 6 presentations in which the first half surface is coarser, 6 presentations in which the second half surface is coarser and 4 in which the two surfaces have the same coarseness. The stimulation parameters were set coherently with gentle touch active exploratory conditions: the indentation force was regulated at 400 mN, whereas the sliding phases were operated at 10 mm/s for 2 s per each half stimulus.

### Psychophysical protocol with naturalistic tactile stimuli

The experiments with naturalistic tactile stimuli were based on a classification task, asking the subjects to identify the stimulus among a set of 4 (paper, rubber, textile, PVC,) or 6 (paper, rubber, textile, PVC, velcro and wood) surfaces^46^.

The stimuli were presented using a mechatronic platform^38^ in randomized sequences of 16 trials for the experiments with 4 surfaces, and in randomized sequences of 24 trials for the experiments with 6 surfaces.

In experimental sessions with ALM and LOP amputee subjects each texture was presented in a passive way to the biomimetic finger (see Fig. 1a) and the resulting spike trains were injected by means of the substitutive intraneural stimulation.

The stimuli were also presented for control to a cohort of 10 healthy subjects (6 females, 4 males, age range [23–34]) following the same passive touch protocol. In this case each texture was sliding while in contact with the index finger of the dominant hand, while the forearm was set in a comfortable position.

In both cases during the sliding phase the speed was fixed at 10 mm/s and the temporal duration of the phase was fixed at 6 s.

### Decoding of naturalistic textures

The behavioral performance of ALM in the decoding of the six naturalistic textures set was compared against decoding of the same set based on the physical properties of the textures and different features of the neuromorphic spike patterns generated by the biomimetic fingertip.

#### Decoding based on physical features

We selected as physical features describing the physical properties of the naturalistic textures three variables based on the forces measured with the load cell (see above): (i) the normal force during the indentation phase; (ii) the friction during slide, measured as the ratio between the normal and tangential force during the horizontal movement of the fingertip; (iii) the roughness, measured as the standard deviation of the normal force during slide. Each trial was described by the average over time of these variables. Prior to decoding, each variable was z-scored over trials. Inter-trial distance was then measured with Euclidean distance in the space of these three variables. Decoding was performed with k Nearest Neighbors method, with k = 5 and standard leave-one-out validation.

#### Decoding based on neural features

To evaluate possible ways that information about naturalistic textures could be extracted from the neuromorphic spike trains injected in the peripheral nerves of the stump, we decoded the identity of the textures starting from the features of the spike trains. First, we followed the two approaches described in our previous study^21^. We evaluated the overall information conveyed by temporal codes with an approach based on precise spike timing, measuring the distance between trials by means of the Victor-Purpura distance between the associated spike trains. This analysis was performed also with the spike trains generated by the population model (see below). We performed also a simpler classification by means of two features directly related to the physical features described in the previous subsection: the spike count and the coefficient of variation (CV) of the inter-spike interval during sliding. Inter-trial distance was defined as Euclidean distance in the two-dimensional space defined by these features. In a further set of analyses a third feature based in physical features was added, i.e., the average spike count during the indentation phase. Inter-trial distance was then defined as Euclidean distance in the three-dimensional space defined by all the features. In all cases decoding was performed according to the defined distance with k Nearest Neighbors method, with k = 5 and standard leave-one-out validation.

### Confusion matrix information

We computed the confusion matrix information associated to subject ALM classification of six naturalistic textures. This consists in the mutual information between the set T of the six presented textures and the set T’ of the decoded textures, given by

6 $$I(T;T{\prime} )=\sum _{t,t{\prime} \in T}P(t)P(t{\prime} |t)lo{g}_{2}\frac{P(t{\prime} |t)}{P(t\text{'})}$$

where *P(t)* is 1/6 by experimental design, *P(t’)* is the probability across all trials of having the answer *t’*, and *P(t’|t)* the conditional probability of answer *t’* when the texture presented is *t*.

We tackled the information bias due to the limited data set^47^ with the following steps:

- we applied the Panzeri-Treves bias correction
- we subtracted the average value of information of 200 bootstrap repetitions.

### Mutual information between neural features and textures

We computed the mutual information between the set T composed by all six textures and the following sets of stimuli features: (i) spike count during indentation, (ii) spike count during sliding, (iii) coefficient of variation of interspike interval during sliding, (iv–vii) all possible combinations of the preceding features.

Mutual information between a set of textures T and a set of stimuli features F is defined as

7 $$I(T;F)=\sum _{t\in T,f\in F}P(t)P(f|t)lo{g}_{2}\frac{P(f|t)}{P(f)}$$

where *P(t)* is 1/6 by experimental design, *P(f)* is the probability across all trials of observing a given value *f* of the feature *F*, and *P(f|t)* the conditional probability of observing the value *f* of the feature *F* in trials in which the texture presented is *t*. In case of multiple-features codes, f is not a scalar but a vector in which the n-th dimension is given by the feature F_n_ [f_1_ ∈ F_1,_ f_2_ ∈ F_2_……].

We tackled the information bias due to the limited data set with the following steps:

- we limited the number of bins of the signal to six (equipopulated), so to have the same dimensionality of the confusion matrix information
- we applied the Panzeri-Treves bias correction
- in case of monodimensional codes we subtracted the average value of information of 200 bootstrap repetitions^47^.
- in case of multidimensional codes, we applied the information shuffling correction^47^.

### Decoding based on a neurophysiological model of mechanoreceptor populations

The six different texture samples were selected also to analyze the decoding performance of a neurophysiological model (*TouchSim*) recently published^14,48^. Texture profiles (obtained by a laser scanner, Gocator 2330 LMI) were used as inputs to the *TouchSim* model (with surfaces approximated as rigid). The scans were repeated 16 times each, to simulate a light form of experimental noise during the acquisition.

The contact area on the first digit fingertip was defined as circular (20 mm^2^), and the resolution (pin spacing) was set to 0.05 mm. The skin contact area moved across the texture at a speed of 10 mm/s for 6 s (as in the experiments). The indentation depth was set to 1 mm at the center of contact and followed a circular profile toward the border of contact. The motion direction was fixed as the one used in the experiments.

### Analysis of results

The 95% confidence intervals for performance estimate for overall and single textures decoding performances were computed for every code with exact Clopper Pearson method (binofit function in Matlab) and compared against chance level (3/8 for gratings, 1/4 or 1/6 for naturalistic textures) to assess performance significance. Parameters for logistic fit of performance as a function of spatial periods for gratings coarseness discrimination were estimated with generalized linear regression (glmfit function in Matlab), then the optimal logistic fit function was generated (glmval function in Matlab) and its accuracy evaluated as the squared correlation coefficient between data and fit.

## Supplementary information

Supplementary information.

## Data Availability

The datasets generated during and/or analyzed during the current study are available from the corresponding author on reasonable request.

## References

[1] Abraira VE, Ginty DD (2013). The sensory neurons of touch. Neuron.

[2] Bensmaïa SJ, Craig JC, Yoshioka T, Johnson KO (2006). SA1 and RA afferent responses to static and vibrating gratings. J Neurophysiol.

[3] Vallbo AB, Johansson RS (1984). Properties of cutaneous mechanoreceptors in the human hand related to touch sensation. Hum Neurobiol.

[4] Hollins M, Risner SR (2000). Evidence for the duplex theory of tactile texture perception. Percept Psychophys.

[5] Yoshioka T, Gibb B, Dorsch AK, Hsiao SS, Johnson KO (2001). Neural coding mechanisms underlying perceived roughness of finely textured surfaces. J Neurosci.

[6] Johansson RS, Flanagan JR (2009). Coding and use of tactile signals from the fingertips in object manipulation tasks. Nature Rev. Neurosci..

[7] Weber AI (2013). Spatial and temporal codes mediate the tactile perception of natural textures. Proc Natl Acad Sci USA.

[8] Tan DW (2014). A neural interface provides long-term stable natural touch perception. Sci Transl Med.

[9] Raspopovic, S. *et al*. Bioengineering: Restoring natural sensory feedback in real-time bidirectional hand prostheses. *Science Translational Medicine* **6**, 10.1126/scitranslmed.3006820 (2014). 10.1126/scitranslmed.3006820 24500407

[10] Dhillon GS, Horch KW (2005). Direct neural sensory feedback and control of a prosthetic arm. IEEE Trans Neural Syst Rehabil Eng.

[11] Graczyk EL, Resnik L, Schiefer MA, Schmitt MS, Tyler DJ (2018). Home Use of a Neural-connected Sensory Prosthesis Provides the Functional and Psychosocial Experience of Having a Hand Again. Sci Rep.

[12] Petrini, F. M. *et al*. Six-months assessment of a hand prosthesis with intraneural tactile feedback. *Ann Neurol*, 10.1002/ana.25384 (2018). 10.1002/ana.25384 30474259

[13] Saal HP, Bensmaia SJ (2015). Biomimetic approaches to bionic touch through a peripheral nerve interface. Neuropsychologia.

[14] Saal HP, Delhaye BP, Rayhaun BC, Bensmaia SJ (2017). Simulating tactile signals from the whole hand with millisecond precision. Proceedings of the National Academy of Sciences.

[15] Black, C., Darie, R. & Borton, D. Organic Electronics for Artificial Touch. *Trends Neurosci*, 10.1016/j.tins.2018.07.010 (2018). 10.1016/j.tins.2018.07.010 30093073

[16] Pfeifer, R. & Bongard, J. *How the body shapes the way we think: a new view of intelligence*. (MIT press, 2006).

[17] Oddo, C. M. *et al*. Intraneural stimulation elicits discrimination of textural features by artificial fingertip in intact and amputee humans. *eLife* **5**, 10.7554/eLife.09148 (2016). 10.7554/eLife.09148 PMC4798967 26952132

[18] Kim Y (2018). A bioinspired flexible organic artificial afferent nerve. Science.

[19] Osborn, L. E. *et al*. Prosthesis with neuromorphic multilayered e-dermis perceives touch and pain. *Science Robotics* **3** (2018). 10.1126/scirobotics.aat3818 PMC7051004 32123782

[20] Boretius T (2010). A transverse intrafascicular multichannel electrode (TIME) to interface with the peripheral nerve. Biosens Bioelectron.

[21] Rongala UB, Mazzoni A, Oddo CM (2017). Neuromorphic artificial touch for categorization of naturalistic textures. IEEE Transactions on Neural Networks and Learning Systems.

[22] Oddo CM (2007). Investigation on calibration methods for multi-axis, linear and redundant force sensors. Measurement Science and Technology.

[23] Delhaye BP, Long KH, Bensmaia SJ (2018). Neural Basis of Touch and Proprioception in Primate Cortex. Compr Physiol.

[24] Panzeri S, Brunel N, Logothetis NK, Kayser C (2010). Sensory neural codes using multiplexed temporal scales. Trends in Neurosciences.

[25] Zuo Y (2015). Complementary contributions of spike timing and spike rate to perceptual decisions in rat S1 and S2 cortex. Curr Biol.

[26] Oddo, C. M. *et al*. Artificial spatiotemporal touch inputs reveal complementary decoding in neocortical neurons. *Scientific Reports* **7**, 10.1038/srep45898 (2017). 10.1038/srep45898 PMC5379202 28374841

[27] Quian Quiroga R, Panzeri S (2009). Extracting information from neuronal populations: information theory and decoding approaches. Nat Rev Neurosci.

[28] Victor JD, Purpura KP (1996). Nature and precision of temporal coding in visual cortex: a metric-space analysis. J Neurophysiol.

[29] Borton D, Micera S, Millán JER, Courtine G (2013). Personalized neuroprosthetics. Sci Transl Med.

[30] Callier, T., Suresh, A. K. & Bensmaia, S. J. Neural Coding of Contact Events in Somatosensory Cortex. *Cereb Cortex*, 10.1093/cercor/bhy337 (2019). 10.1093/cercor/bhy337 PMC6917522 30668644

[31] Pei YC, Bensmaia SJ (2014). The neural basis of tactile motion perception. J Neurophysiol.

[32] Bologna, L. L. *et al*. A closed-loop neurobotic system for fine touch sensing. *Journal of Neural Engineering* **10** (2013). 10.1088/1741-2560/10/4/046019 23883543

[33] Bologna LL, Pinoteau J, Brasselet R, Maggiali M, Arleo A (2011). Encoding/decoding of first and second order tactile afferents in a neurorobotic application. Journal of Physiology-Paris.

[34] Pack CC, Bensmaia SJ (2015). Seeing and Feeling Motion: Canonical Computations in Vision and Touch. PLoS Biol.

[35] Rongala, U. B. *et al*. Tactile Decoding of Edge Orientation With Artificial Cuneate Neurons in Dynamic Conditions. *Frontiers in Neurorobotics* **13**, 10.3389/fnbot.2019.00044 (2019). 10.3389/fnbot.2019.00044 PMC6614200 31312132

[36] Valle G (2018). Biomimetic Intraneural Sensory Feedback Enhances Sensation Naturalness, Tactile Sensitivity, and Manual Dexterity in a Bidirectional Prosthesis. Neuron.

[37] Beccai L (2005). Design and fabrication of a hybrid silicon three-axial force sensor for biomechanical applications. Sensors and Actuators A: Physical.

[38] Oddo CM (2011). A mechatronic platform for human touch studies. Mechatronics.

[39] Oddo CM (2011). Roughness Encoding in Human and Biomimetic Artificial Touch: Spatiotemporal Frequency Modulation and Structural Anisotropy of Fingerprints. Sensors.

[40] Izhikevich EM (2003). Simple model of spiking neurons. Neural Networks, IEEE Transactions on.

[41] Spigler, G., Oddo, C. M. & Carrozza, M. C. In *2012 4th IEEE RAS and EMBS International Conference on Biomedical Robotics and Biomechatronics*, *BioRob 2012*. 1913–1918 (2012).

[42] Oddo CM (2007). Investigation on calibration methods for multi-axis, linear and redundant force sensors. Meas Sci Technol.

[43] Kim SS, Sripati AP, Bensmaia SJ (2010). Predicting the timing of spikes evoked by tactile stimulation of the hand. Journal of neurophysiology.

[44] Carboni, C. *et al*. In *2017**IEEE Biomedical Circuits and Systems Conference (BioCAS)*. 1–4 (2017).

[45] Bisoni L, Carboni C, Raffo L, Carta N, Barbaro M (2015). An HV-CMOS Integrated Circuit for Neural Stimulation in Prosthetic Applications. IEEE Transactions on Circuits and Systems II: Express Briefs.

[46] Norwich KH (1981). The magical number seven: making a “bit” of “sense”. Percept Psychophys.

[47] Magri C, Whittingstall K, Singh V, Logothetis NK, Panzeri S (2009). A toolbox for the fast information analysis of multiple-site LFP, EEG and spike train recordings. BMC Neurosci.

[48] Okorokova EV, He Q, Bensmaia SJ (2018). Biomimetic encoding model for restoring touch in bionic hands through a nerve interface. J Neural Eng.

