# Author Correction: Morphological Neural Computation Restores Discrimination of Naturalistic Textures in Trans-radial Amputees

**DOI:** 10.1038/s41598-021-96290-y

**Published:** 2021-08-11

**Authors:** Alberto Mazzoni, Calogero M. Oddo, Giacomo Valle, Domenico Camboni, Ivo Strauss, Massimo Barbaro, Gianluca Barabino, Roberto Puddu, Caterina Carboni, Lorenzo Bisoni, Jacopo Carpaneto, Fabrizio Vecchio, Francesco M. Petrini, Simone Romeni, Tamas Czimmermann, Luca Massari, Riccardo di Iorio, Francesca Miraglia, Giuseppe Granata, Danilo Pani, Thomas Stieglitz, Luigi Raffo, Paolo M. Rossini, Silvestro Micera

**Affiliations:** 1 The Biorobotics Institute, Scuola Superiore Sant’Anna, Pisa, Italy; 2 Department of Excellence in Robotics & A.I, Scuola Superiore Sant’Anna, Pisa, Italy; 3 Department of Electrical and Electronic Engineering, Università di Cagliari, Cagliari, Italy; 4 Brain Connectivity Laboratory, IRCCS San Raffaele Pisana, Roma, Italy; 5 Bertarelli Foundation Chair in Translational Neuroengineering, Centre for Neuroprosthetics and Institute of Bioengineering, School of Engineering, École Polytechnique Fédérale de Lausanne (EPFL), Lausanne, Switzerland; 6 Department of Health Sciences and Technology, Institute for Robotics and Intelligent Systems, ETH Zürich, Zürich, Switzerland; 7 Institute of Neurology, Catholic University of The Sacred Heart, Policlinic A. Gemelli Foundation, Roma, Italy; 8 Laboratory for Biomedical Microtechnology, Department of Microsystems Engineering–IMTEK; Bernstein Center Freiburg and BrainLinks-BrainTools Center, University of Freiburg, Freiburg, Germany

Correction to: *Scientific Reports* 10.1038/s41598-020-57454-4, published on 16 January 2020

In the original version of this Article Paolo M. Rossini was incorrectly affiliated with ‘Institute of Neurology, Catholic University of The Sacred Heart, Policlinic A. Gemelli Foundation, Roma, Italy’. The correct affiliation is listed below.

Brain Connectivity Laboratory, IRCCS San Raffaele Pisana, Roma, Italy.

The original Article has been corrected.

