# Supplementary material for: Morphological Neural Computation Restores Discrimination of Naturalistic Textures in Trans-radial Amputees
Source: Sci Rep. 2020 Jan 16;10:527. doi: 10.1038/s41598-020-57454-4 (PMC6965126; doi:10.1038/s41598-020-57454-4)
Supplement: Supplementary file 1 — Supplementary information. [file 41598_2020_57454_MOESM1_ESM.docx]

**Morphological neural computation restores discrimination of naturalistic textures in trans-radial amputees**

Alberto Mazzoni^1,2^ †, Calogero M. Oddo^1,2^ †, Giacomo Valle^1,2^, Domenico Camboni^1,2^, Ivo Strauss^1,2^, Massimo Barbaro^3^, Gianluca Barabino^3^, Roberto Puddu^3^, Caterina Carboni^3^, Lorenzo Bisoni^3^, Jacopo Carpaneto^1,2^, Fabrizio Vecchio^4^, Francesco M. Petrini^5,6^, Simone Romeni^1,2^, Tamas Czimmermann^1,2^, Luca Massari^1,2^, Riccardo di Iorio^7^, Francesca Miraglia^4^, Giuseppe Granata^7^, Danilo Pani^3^, Thomas Stieglitz^8^, Luigi Raffo^3^, Paolo M. Rossini^4^, Silvestro Micera^1,2,5*^

^1^The Biorobotics Institute, Scuola Superiore Sant’Anna, Pisa, Italy

^2^Department of Excellence in Robotics & A.I., Scuola Superiore Sant’Anna, Pisa, Italy

^3^Department of Electrical and Electronic Engineering, Università di Cagliari, Cagliari, Italy

^4^Brain Connectivity Laboratory, IRCCS San Raffaele Pisana, Roma, Italy

^5^Bertarelli Foundation Chair in Translational Neuroengineering, Centre for Neuroprosthetics and Institute of Bioengineering, School of Engineering, École Polytechnique Fédérale de Lausanne (EPFL), Lausanne, Switzerland

^6^Department of Health Sciences and Technology, Institute for Robotics and Intelligent Systems, ETH Zürich, Zürich, Switzerland

^7^Institute of Neurology, Catholic University of The Sacred Heart, Policlinic A. Gemelli Foundation, Roma, Italy

^8^Laboratory for Biomedical Microtechnology, Department of Microsystems Engineering–IMTEK; Bernstein Center Freiburg and BrainLinks-BrainTools Center University of Freiburg, Freiburg, Germany

†These authors share first authorship based on equal contribution

*Correspondence to: [silvestro.micera@santannapisa.it](mailto:silvestro.micera@santannapisa.it) [@epfl.ch], [calogero.oddo@santannapisa.it](mailto:calogero.oddo@santannapisa.it)

**SUPPLEMENTARY MATERIALS**


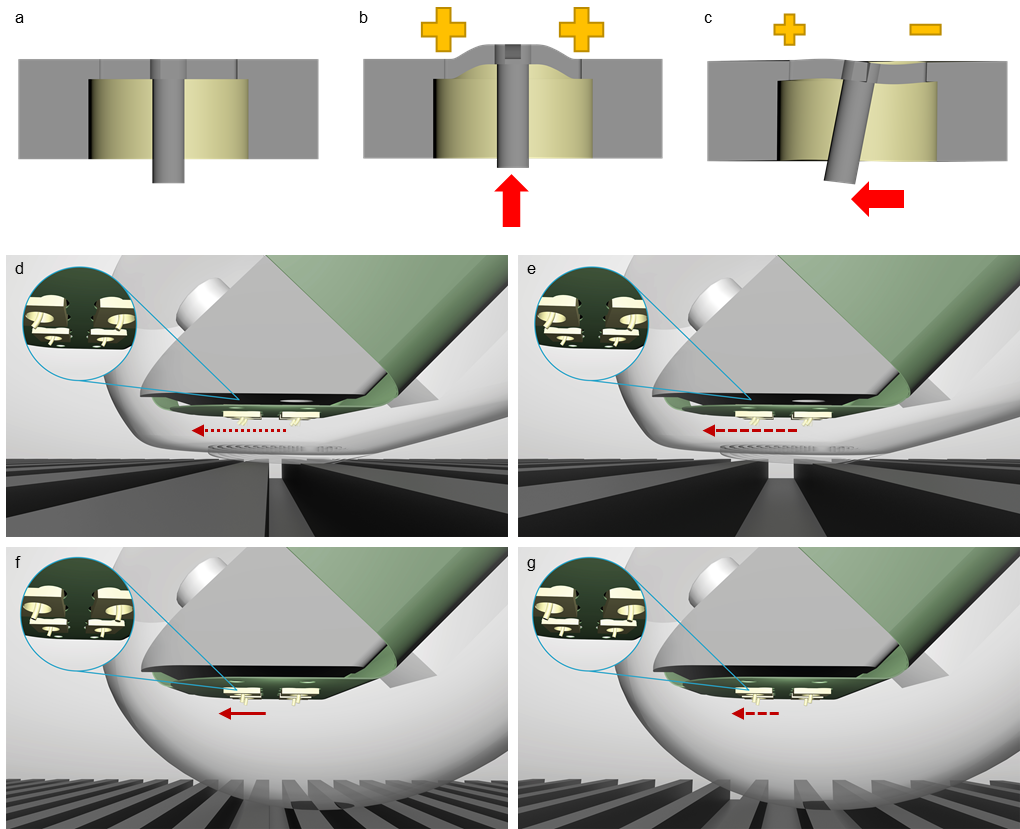


**Supplementary Figure 1** | **a-c.** Illustration of mechanostransduction operated by the used MEMS tactile sensor: no load condition **(a)**, normal load condition **(b)** and tangential load condition **(c)**. **d-g.** Illustration of the shear stress (red arrow) due to the application of fine gratings **(d, f)** and coarse gratings **(e, g)**, transduced by type-1 surface-located **(d, e)** and type-2 deeply-located **(f, g)** artificial receptors. Type-1 receptors are able to encode the spatial characteristics of both fine **(d)** and coarse stimuli **(e)** by means of their temporal response, whereas type-2 receptors, due to their deeper positioning, are subject to a low-pass spatial filtering that allows the temporal encoding of the spatial characteristics of coarse gratings **(g)** but not of fine ones **(f)**.


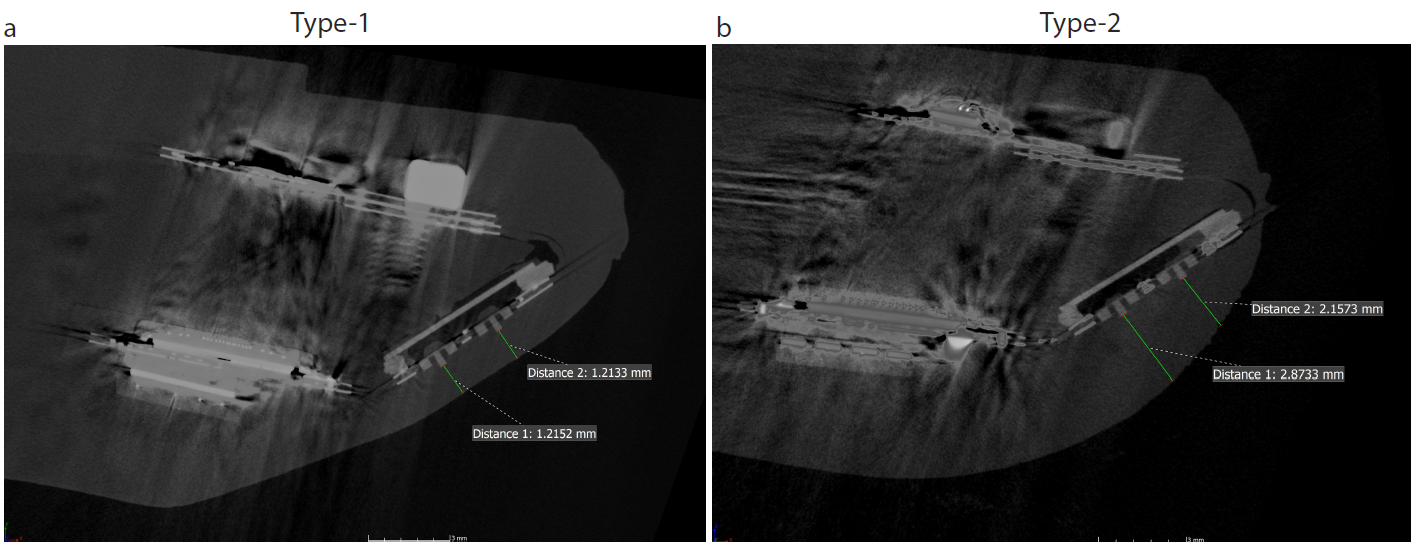


Supplementary Figure 2 | Tomographic images of the biomimetic fingertip with sensor location mimicking location of type-1 **(a)** and type-2 **(b)** mechanoreceptors.


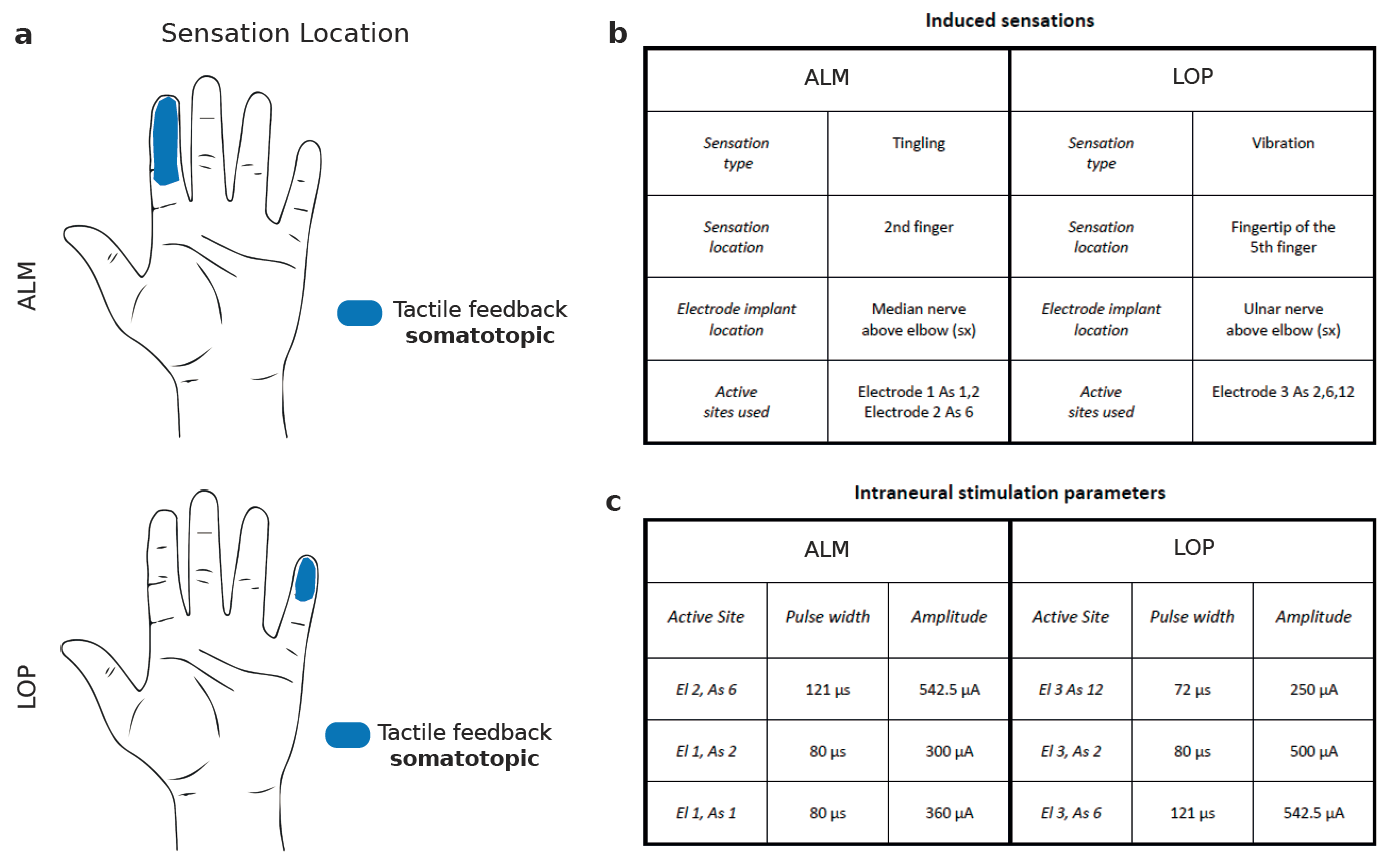


Supplementary Figure 3 | Locations of the induced tactile sensations in two trans-radial amputees with stimulation of median or ulnar nerve. a. Mapping of locations induced in the phantom hands of two amputees. b. Induced sensations in the amputees. c. Stimulation parameters.


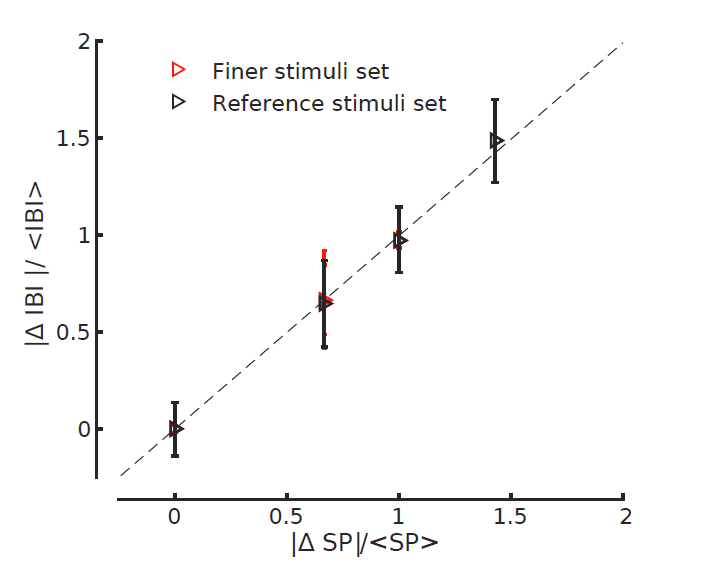


Supplementary Figure 4 | Relationship between relative variations of inter burst interval and relative variation of spatial period. Black markers indicate reference stimuli set and red markers finer stimuli set. Black dashed line indicates linear fit.


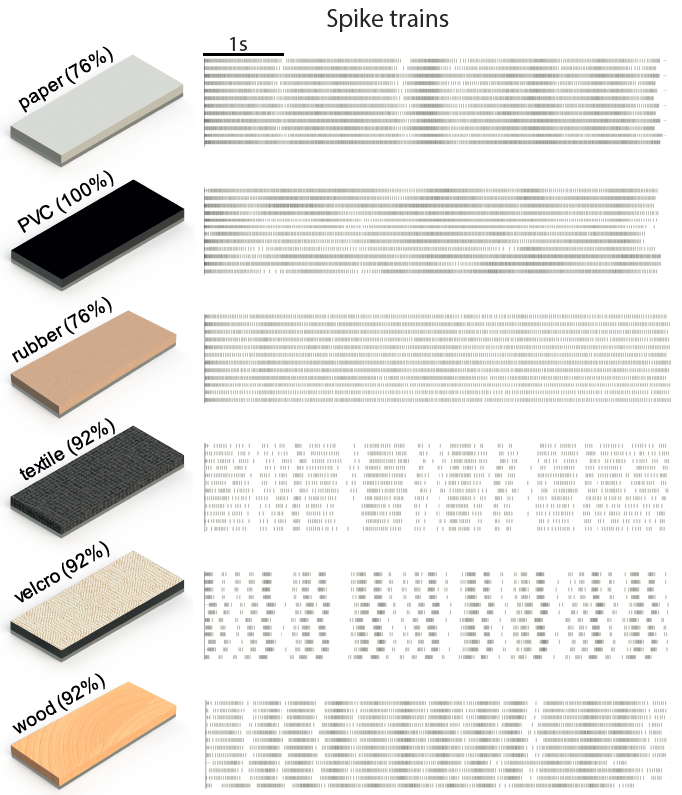


Supplementary Figure 5 | Spike trains transduced by the biomimetic finger with type-1 artificial receptors. Left: Picture of the stimuli used in the naturalistic texture identification task. Above each texture the average decoding performance of subject ALM in the six-textures classification trial is reported. Right: Full set of spike trains generated by the sliding over the different surfaces (6 seconds, 12 trials).


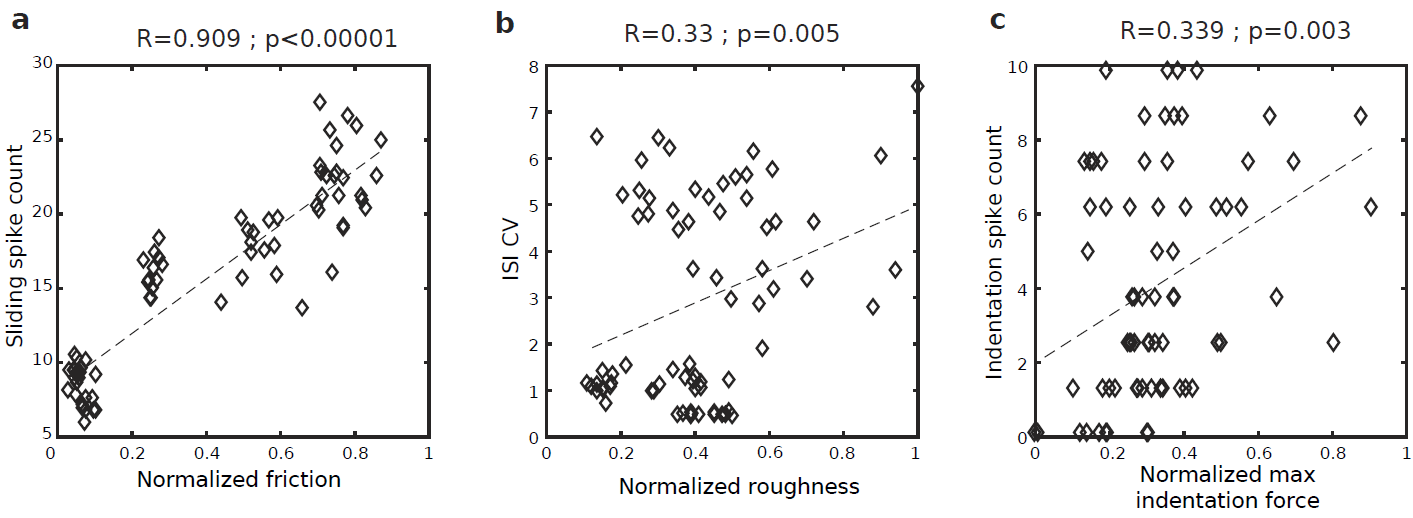


Supplementary Figure 6 | Relationship between physical features of the textures and firing activity. a. Average spike count as a function of friction during sliding for the different textures. b. Coefficient of variation of the inter-spike interval as a function of texture roughness during sliding for the different textures. c. Average spike count as a function of maximal normal force during indentation for the different textures. In each panel title reports Pearson correlation coefficient and significance.


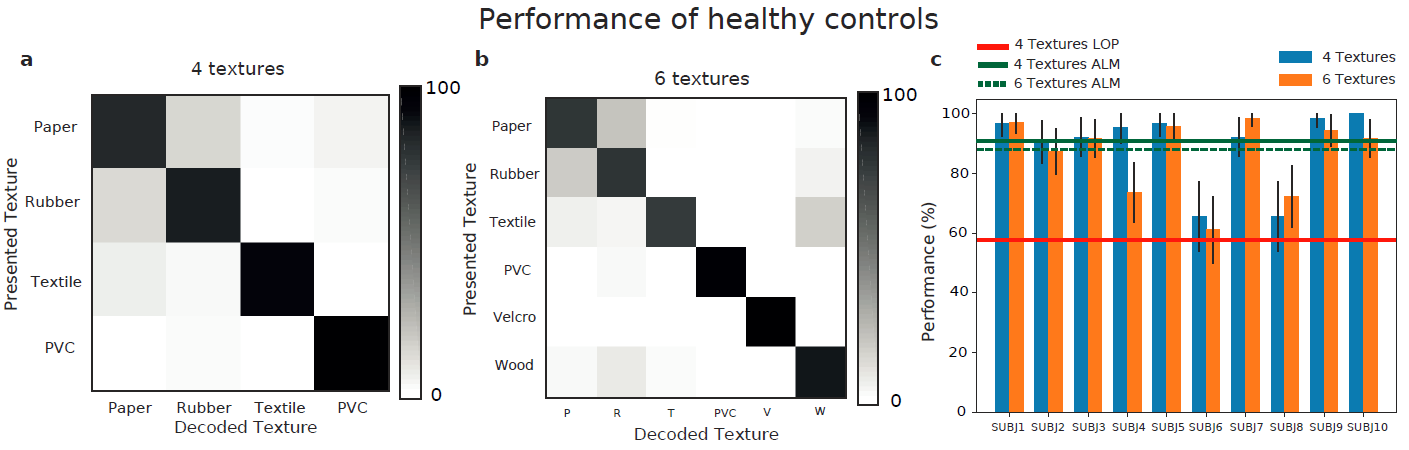


Supplementary Figure 7 | Control psychophysical experiments with intact subjects perceiving the tactile stimuli naturally with passive stimulation of fingertip (see Methods). a. Results of experiments with 4 stimuli; from left to right: confusion matrix (same as Figure 3a), texture-wise results (same as Figure 3b) averaged over all subjects, cumulative distribution of overall performance across subjects (red line shows distribution fit, horizontal lines indicate performance of ALM and LOP). b. Same as a with results of experiments with 6 stimuli.


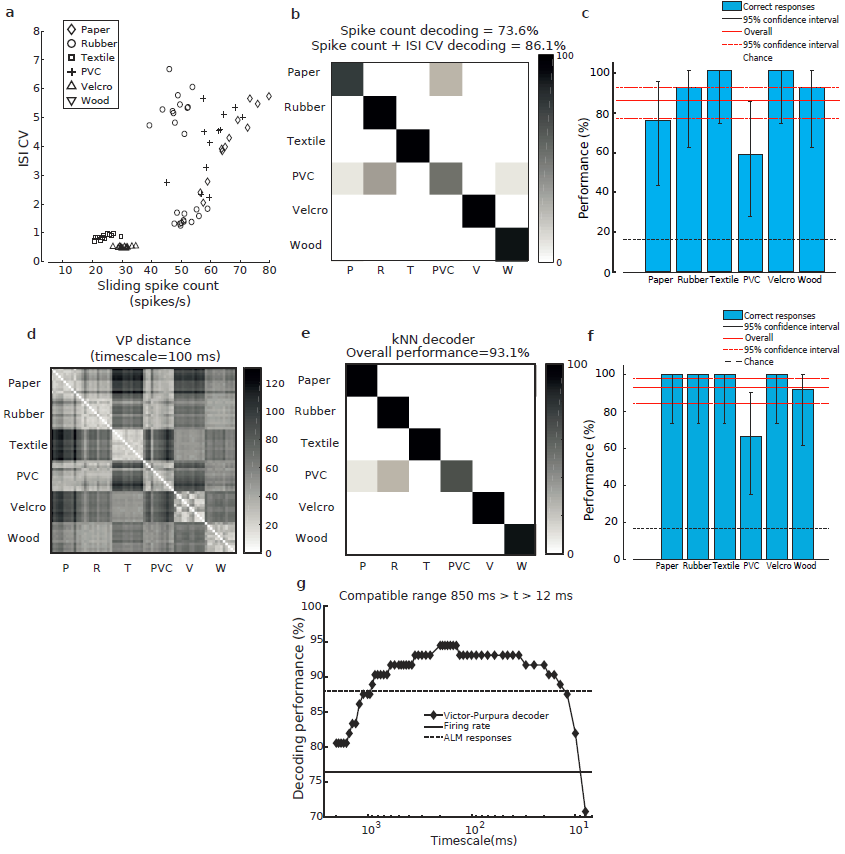


**Supplementary Figure 8** | **a.** Scatter plot of sliding spike count and coefficient of variation of the inter-spike interval for each presentation of each of the naturalistic textures. b. Confusion matrix of kNN decoding of textures based on the aforementioned features. c. Average performance and confidence for single-texture and overall decoding. **d.** Confusion matrix related to Victor-Purpura spike train-distance (with a timescale of 100 ms) for all the naturalistic stimuli. e. Confusion matrix of kNN decoding of textures based on the aforementioned features. f. Average performance and confidence for single-texture and overall decoding. g. Decoding performances based on Victor Purpura spike train-distance over a broad range of timescales.


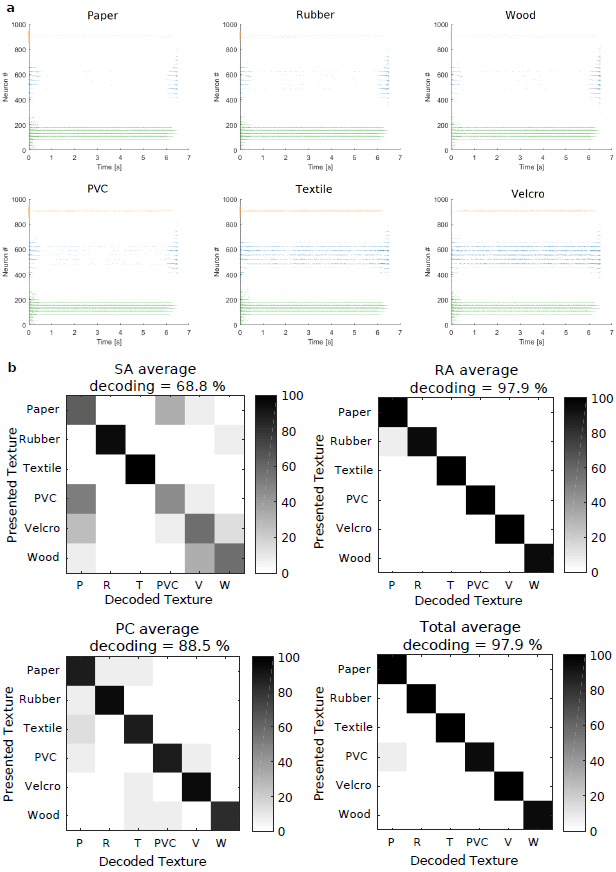


**Supplementary Figure 9** | **a.** Single trial raster plots of SA (green), RA (cyan), PC (orange) mechanoreceptors during the presentation of different stimuli (see panel titles). **b.** Confusion matrices for Victor-Purpura distance-based decoding of textures from population activity of different mechanoreceptors.

Supplementary Table 1 | Spatial periods of the gratings used in the experiments. a. Reference set of stimuli. b. Finer set of stimuli.

Supplementary Table 1a. Reference set of grating stimuli.

| SP1 (mm) | SP2 (mm) | ΔSP (mm) | ΔSP/<SP> |
| --- | --- | --- | --- |
| 1.5 | 1.5 | 0.0 | 0.00 |
| 1.0 | 2.0 | 1.0 | 0.66 |
| 1.0 | 3.0 | 2.0 | 1.00 |
| 0.5 | 3.0 | 2.5 | 1.43 |

Supplementary Table 1b. Finer set of grating stimuli.

| SP1 (mm) | SP2 (mm) | ΔSP (mm) | ΔSP/<SP> |
| --- | --- | --- | --- |
| 1.5 | 1.5 | 0.0 | 0.00 |
| 0.5 | 1.0 | 0.5 | 0.66 |
| 1.0 | 2.0 | 1.0 | 0.66 |
| 1.0 | 3.0 | 2.0 | 1.00 |
